# Perception of Special-Care Dentistry among Dental Students at Universitas Indonesia: A Cross-Sectional Study

**DOI:** 10.3390/dj12010019

**Published:** 2024-01-22

**Authors:** Atik Ramadhani, Fiki Rizqa Izzati, Diah Ayu Maharani, Normaliza Ab Malik, Febriana Setiawati

**Affiliations:** 1 Department of Dental Public Health and Preventive Dentistry, Faculty of Dentistry, Universitas Indonesia, Depok 16424, Indonesia; atikramadhani@ui.ac.id (A.R.); fiki.rizqa@ui.ac.id (F.R.I.); diah.ayu64@ui.ac.id (D.A.M.); 2 Department of Conservative Dentistry and Prosthodontic, Faculty of Dentistry, Universiti Sains Islam Malaysia, Bandar Baru Nilai, Nilai 71800, Malaysia; liza_amalik@usim.edu.my

**Keywords:** dental education, perception, disabled persons, special-care dentistry

## Abstract

Objective: To assess the perceptions of special-care dentistry (SCD) among dental students in the Faculty of Dentistry, Universitas Indonesia, Indonesia. Methods: This cross-sectional study used a self-administered online questionnaire, and all preclinical and clinical students were invited to participate. The survey items comprised four domains related to participants’ characteristics, perceptions of SCD, perceptions of managing patients with special needs, and perceptions of SCD education. The chi-square test and Mann–Whitney test were used in the analysis. Results: A total of 572 students participated in this study. The findings showed that the clinical students were more familiar with SCD than the preclinical students were (*p* = 0.008). A statistically significant relationship was found between the study program and the ability to work independently with special-needs patients after graduation (*p* < 0.001), the ability to refer special-needs patients to specialists (*p* = 0.042), the perception of postgraduate training-program needs (*p* < 0.001), and the opportunity to consider postgraduate training (*p* = 0.004). Conclusion: Most of the respondents had a favorable perception of SCD. Thus, an improved SCD curriculum and SCD training for undergraduate and postgraduate students should be provided to develop the knowledge and skills needed to provide care to special-needs patients.

## 1. Introduction

It is estimated that more than one billion people live with a disability. Approximately 15% of the world’s population, up to 190 million adults and 93 million children under 15 years of age, experience significant limitations in functioning or even suffer from severe disability. This number is projected to increase, partly due to an aging population and complex health problems [1]. These people are more likely to develop oral disease due to obstacles that prevent them from obtaining adequate personal and professional oral health [2,3]. They tend to choose treatments and services full of empathy, professionalism, and compassion, emphasizing the need for enhanced intervention to provide preventive, curative, and long-term oral-health care [4]. Dentists’ awareness and positive attitudes toward people with disabilities can improve oral-health services [5].

Treating patients with special needs has many challenges. The nonavailability of dentists is reported to be a significant barrier to accessing dental care services [6]. Previous studies have reported that some dentists are hesitant to serve patients with special needs, which leads to an increase in oral disease burden and unmet treatment needs. They have a perceived lack of knowledge and confidence in their ability to treat special-needs patients due to insufficient training, experience, and difficulty in obtaining patient information [4,7]. In addition, barriers within the public dental system, such as inadequate funding, facilities, and equipment, as well as poor processes and policies, prevent dentists from being able to provide the care that these patients need [6].

The challenge of treating patients with special needs prompted the development of special-care dentistry (SCD). Special-care dentistry is an oral-health service for those whose physical, mental, developmental, or cognitive conditions prevent them from receiving regular dental treatment [8]. A previous study reported good perceptions of the experiences of students who attended didactic learning and clinical training in SCD, which could improve access to oral-health services and the status of oral health for patients with special needs. The students had situated learning that emphasized confidence, awareness, and experience throughout the fieldwork visit [9]. However, many dental schools still do not provide adequate training for their students to care for special-needs patient groups, resulting in a limited number of dentists specializing in SCD and a lack of interprofessional collaboration for specialized services [6,10].

In 2018, based on the National Socioeconomic Survey (Survei Sosial Ekonomi Nasional/Susenas) by the Central Bureau of Statistics (Badan Pusat Statistics/BPS), the total population of people with disabilities in Indonesia was 30.38 million people (14.2% of the total population) [11]. According to the National Basic Health Survey (RISKESDAS), the proportion of individuals with disabilities aged 18–59 years was 22% [12]. However, Indonesia faces many obstacles and challenges in accessing healthcare for people with disabilities. A previous study regarding the perception of SCD among dental professionals in Jakarta, the capital city of Indonesia, showed that most respondents did not have an SCD education during their undergraduate dental-school training and, thus, did not provide treatment to special-needs patients. Although most respondents reported having a poor perception of SCD, they were generally motivated and interested in SCD training [13].

Therefore, this study aimed to determine the perception of SCD among dental students at Universitas Indonesia. This information would provide feedback and an analysis of undergraduate students’ interest in, acceptance of, and growth in SCD, which may impact the development of SCD curricula in Indonesian dental schools.

## 2. Materials and Methods

### 2.1. Study Design, Setting, and Participants

A cross-sectional study using a self-administered online questionnaire was carried out in October 2021 at the Faculty of Dentistry, Universitas Indonesia. The present study followed the recommendations of the Strengthening the Reporting of Observational Studies in Epidemiology (STROBE) statement [14]. Ethical approval for this study was obtained from the Dental Research Ethics Committee, Faculty of Dentistry, Universitas Indonesia (Protocol Number: 010350721). This study was conducted in full accordance with the Declaration of Helsinki. All dental students who were in preclinical and clinical programs at Universitas Indonesia (*n* = 813) were invited to participate in the survey. The link to the Google Form was distributed through social media via WhatsApp and Line messaging, and all participants signed up voluntarily. Informed consent was obtained from all the students participating in this study, and the participants’ responses were kept confidential. The results with errors in the information record were excluded.

### 2.2. Questionnaire Survey

The English-language questionnaire was adapted from an existing validated instrument from a previous study [15]. Each item in the questionnaire was reviewed by a panel expert to ensure its relevance, clarity, and comprehensiveness. Some items were modified during the pilot study. The final survey items consisted of four domains: (1) participants’ characteristics; (2) perception of managing patients with special needs; (3) perception of SCD; and (4) perception of SCD education. A total of 19 questions were included in the final survey. The participants’ characteristics consisted of two items: gender and study program. If the student was in a clinical program, two follow-up questions were asked to assess his or her experience in treating patients with special needs and what patient category they had treated. One question is how to determine the ability of participants to treat special-needs patients upon graduation. The perception of SCD consisted of four questions about the definition, awareness, terminology, and prior knowledge of SCD. The perception of managing patients with special needs consisted of one question using a five-point Likert scale that focused on the perception of comfort: 1 (very uncomfortable), 2 (uncomfortable), 3 (neutral), 4 (comfortable), or 5 (very comfortable) in treating special-needs patients. One question concerned the criteria that would be considered when referring patients to a general or SCD specialist: 1 (never), 2 (almost never), 3 (sometimes), 4 (often), or 5 (always). A higher score indicated a more favorable perception of treating and referring special-needs patients. Finally, the perception of SCD education included eight questions. One question assessed the perception of current student training in SCD. If the students received clinical training in managing special-needs patients, one follow-up question was asked to evaluate the adequacy of training in preparing students to treat special-needs patients following graduation. There were two additional questions, each concerning didactic teaching, clinical teaching, and postgraduate training.

### 2.3. Validity and Reliability of the Questionnaire

A total of 12 students from the preclinical program (not participating in this study) were surveyed with the questionnaire in a pilot study. The participants were required to answer and evaluate the questionnaire in terms of its clarity, readability of the wording, and the feasibility of completing the questionnaire in a reasonable time frame. All the items on the questionnaire were clear and meaningful to the participants after minor modifications were made to some items. In addition, participants needed an average of 10 min to complete the questionnaire. A pilot study to test the reliability of the “managing patients with special needs” questionnaire was conducted among 80 clinical students using Cronbach’s alpha [16], the results of which were not included in the main study. The Cronbach’s alpha values for experience in treating patients with special needs, ability to treat patients with special needs upon graduation, and referring patients to a general or SCD specialist domain were 0.83, 0.89, and 0.7, respectively; therefore, the item was reliable.

### 2.4. Statistical Analysis

The data were entered into Microsoft Excel 2010 and processed using the IBM SPSS 26.0 statistical software (IBM Corp., Armonk, NY, USA). Descriptive analyses, including percentages, means, and standard deviations, were used to describe the characteristics of the study participants. When the data distribution was not normal, a Mann–Whitney test was performed to assess differences in participants’ perceptions of special-needs patients (study program and clinical-student experience in caring for patients with special needs). Chi-square analysis was used to assess the relationship between the study program and clinical-student experience in caring for special-needs patients on the outcome survey on the perception of and the training program for SCD. The level of statistical significance for all the tests was set at *p* < 0.05 with a confidence interval of 95%.

## 3. Results

From a total of 813 dental students who were invited to participate in this study, 572 respondents completed the questionnaire (a 70% response rate). Table 1 presents the respondents’ characteristics, perception of, and training programs for SCD. There were six times more female students than male students, and most participants were preclinical students (65%). Eighty students (40%) in the clinical education program provided care to a group of patients with special needs, with the highest proportion of special-needs patients being older adults (77.5%). Most respondents were able to define SCD (71.3%) and were aware of the existence of SCD (55.1%). More than one-third of the respondents preferred to use the term special-care dentistry (43.7%) rather than any other term, and they gained knowledge of SCD through the internet (24%). The students’ perceptions of their ability to treat patients with special needs upon graduation varied greatly. Only 31.6% (agree and strongly agree) of the respondents felt confident that they could work independently with special-needs patients upon graduation.

Most respondents reported that they had never received training for SCD (93.9%). Most respondents needed SCD-related didactic learning (95.6%) in their fourth year of education (69.6%). Furthermore, 93.9% of all respondents felt that they should receive clinical training regarding SCD, and most thought that this should occur in the sixth year (72.6%). It is necessary to receive postgraduate SCD training in a dental education program (89.7%), and respondents want to participate in postgraduate SCD training if they have the opportunity (82%) (Table 1).

The students’ perceptions of providing treatment for special-care patients in different categories are summarized in Table 2. Most of the respondents felt comfortable treating patients with visual impairment (3.7 ± 0.8), while they were less comfortable treating patients with behavioral or psychological problems (3.1 ± 0.1). The respondents’ perceptions of referring special-needs patients to specialists are shown in Table 3. Only 12.1% of respondents cited that they would “always” refer to a specialist when they needed a second opinion and did not have appropriate treatment facilities (19.4%).

Table 4 shows the results of students’ perceptions of SCD related to the study program and clinical-student experience with special-needs patients. The relationship between the perception of special-needs patients and the study program showed that there was a significant difference between the two groups regarding the ability to work independently with special-needs patients following graduation (*p* = <0.001) and when they would refer to a specialist (*p* = 0.042). The clinical students were more familiar with SCD than the preclinical students were (*p* = 0.008). In terms of education in SCD related to the study program, there was a significant difference in the perception of postgraduate training-program needs (*p* = <0.001) and the opportunity to consider postgraduate training (*p* = 0.004). These results showed that the preclinical students were more concerned with postgraduate training in SCD than the clinical students were. There was no significant association between the clinical-student experience in caring for special-needs patients or gender and the SCD variables.

## 4. Discussion

Several studies have reported that special-needs patients have a higher prevalence of oral diseases [3]. The prevalence of people with disabilities in Indonesia aged 35–44 years increased from 7.9% in 2013 [17] to 20.3% in 2018 [12]. Dentists need to understand the role they can play in treating these people. More effort should be made to enhance dental education and to increase SCD awareness among dental students [18]. To our knowledge, this was the first study in Indonesia that attempted to investigate dental students’ perceptions of SCD. As a result, dental students need a good understanding of SCD and specific modules should be offered at the undergraduate and clinical levels.

The study’s findings showed that slightly more than half of the respondents claimed they were aware of SCD (55.1%). This result was lower than that in Malaysian dental students (84.4%) [15] but higher than that in Australian dental students (46.5%) [19]. Increasing awareness may be critical for developing more positive attitudes and professional behavior in managing SCD [9]. Students’ attitudes toward special-needs patients may be altered because of rigorous clinical exposure under adequate supervision, personal experience, societal impact, more community visits, and individual engagement [9,20].

Educational experience in SCD at the undergraduate level is significantly correlated with professional behavior, personal attitudes, and comfort associated with caring for people with disabilities [21,22,23]. Dentists without adequate training and clinical experience reported discomfort with and resistance to caring for special-needs patients [13,24]. This finding is consistent with the results of this study, which showed that only 31.6% (agree and strongly agree) of respondents were confident in working independently with special-needs patients, and almost all students claimed that they had never received clinical training in this area (93.9%). Furthermore, most clinical students (60%) reported that they had never treated special-needs patients. This result is in line with a previous study conducted among Indonesian dentists; the majority of respondents (65.2%) reported that they did not receive SCD education during their undergraduate studies and did not provide treatment for special-needs patients [13]. These findings can be explained by the lack of didactic and clinical (i.e., experiential) learning opportunities related to SCD in dental-education curricula [13,22]. The curriculum for managing individuals with special needs has been found to help increase dental students’ knowledge, skills, attitudes, and level of critical thinking in dealing with people with disabilities [10,25,26]. However, a specific module on SCD has not been established at the Faculty of Dentistry, Universitas Indonesia. Therefore, it is essential to include the topic of SCD as an integral part of dental education curricula.

In the present study, most of the respondents felt that they needed didactic teaching (95.6%) to be provided in the fourth year (69.6%) and clinical training for SCD (93.9%) in the sixth year of their studies (72.6%). Furthermore, they were willing to undertake postgraduate training if they had the opportunity (82%). Adequate clinical experience and good knowledge are essential for establishing clinician competency in treating special-needs patients to provide safe and effective patient care [9,13,27,28]. Formal education or continuing education through special-needs dental workshops for health professionals and students may affect their willingness to treat patients in the future and actively affect patient care [15,24,29]. Thus, it is necessary to have a special comprehensive curriculum regarding the didactic and clinical learning of SCD at the undergraduate and postgraduate levels [23,30]. At the University of Malaya, Malaysia, the SCD curriculum has been integrated into a module for undergraduate students in their final two academic years to provide students with adequate knowledge, clinical skills, attitudes, and self-confidence in caring for special-needs patients [22]. Previous research has shown that postgraduate dental-resident education focused on older adults’ mentation concerns increased confidence and competence in dental care and oral health [30]. This system could also be applied in Indonesia, allowing Indonesian dental students to learn about SCD and to become competent oral-care providers for special-needs patients.

The patient category most frequently treated by clinical students was the elderly (77.5%). This result is in accordance with the findings of a previous study in which more than half of the dentists in DKI Jakarta felt comfortable (70.4%) or positive (61.2%) about treating elderly patients [13]. At the undergraduate level, geriatric dentistry (GD) is a curricular requirement in the majority of dental schools [31]; thus, students have educational experience in this area, and it is significantly associated with professional behavior, attitudes, and comfort in caring for special-needs patients [9,23]. The students’ positive attitudes can be explained by the exposure to information about how to treat this population, the opportunity to interact personally, and getting to know them better, which helped reduce feelings of fear and insecurity [9]. In contrast, the lack of clinical exposure in the treatment of patients with infectious diseases, psychological or behavioral problems, and intellectual disability conditions is reflected in the low level of comfort among dental students toward providing care for those patients. A possible explanation is that the students did not receive enough didactic exposure, and clinical experience indicated discomfort and reluctance to treat special-needs patients [21,32]. Other factors that contribute to the limited treatment for special-needs patients include the time needed to treat the patient, lack of support for the patient, the inability to communicate with patients, and fear of causing harm [32].

Comfort levels and positive attitudes among dental students in treating dental patients with special needs are frequently related to prior clinical exposure [9]. Our results showed that most of the respondents felt comfortable treating patients with visual impairment (3.7 ± 0.8). This may be due to the presence of an assistance escort, such as a family member, who usually accompanies the patient with visual impairment. Similarly, more than half of the dentists in DKI Jakarta felt comfortable treating patients with physical disabilities (67.6%) [13]. More research is needed to investigate the factors that could contribute to the study’s findings. Many respondents in this study would like a second opinion (12.1%) when they treat special-needs patients. Several factors associated with the willingness to treat individuals with special needs included the ongoing support from and communication with specialists in SCD through a structured network [33]. However, in Indonesia, there is currently no or only a very limited treatment pathway for managing special-needs patients for dentists to follow.

Our study revealed a significant difference between preclinical and clinical education programs in terms of the perception of working independently with special-care patients following graduation and the assumption of knowledge about SCD. Clinical students had more knowledge and awareness about SCD and were better able to work independently with special-needs patients following graduation than preclinical students were. This may be because they may have been exposed to cases of SCD in the clinic and because of the complexity of the situation. Didactic-based education, such as lectures, seminars, case assignments, and clinical training in SCD, is needed by students to avoid unpreparedness in caring for special-care patients after they graduate [26]. Moreover, clinical training also plays an important role in ensuring that dental students gain the confidence and level of comfort they need to be willing to provide care to special-care patients in future practice [34,35].

According to the Commission on Dental Accreditation (CODA), one of the current challenges is whether dental students are sufficiently prepared to treat special-care patients after graduation. A new standard should be developed to ensure that dental school graduates are competent in assessing the treatment needs of patients receiving special care [36]. Our results also showed that preclinical and clinical students believe that a dental education program should include a postgraduate training program for SCD. They considered participating in postgraduate training if they had the opportunity. Moreover, clinical students reported needing to refer special-needs patients to specialists more often than preclinical students did. A previous study showed that most dentists in Indonesia have difficulty treating special-care patients because they do not have the special training or education to handle this group of patients. Patients with special needs are always referred to a specialist if a second opinion is needed [13].

As professionals, we have to treat this group of special-care patients with dignity and respect so that they can have equal access to the healthcare system. However, it has been reported that there are barriers to access [35]. The ability of dentists to provide SCD care will reduce the problem of accessing care for special-care patient groups [15,37]. With an increasing number of dentists who are willing and ready to treat special-care patients, the gap in oral-health care that is not being met can be reduced, thereby improving the quality of life of this group of patients [13].

Several limitations were encountered in this study. First, selection bias may be present because most of the respondents were female. This is because the number of male dental students at Universitas Indonesia is low, accounting for only 10–15% of the total number of dental school students each year. Further studies on potential barriers to treating special-needs patients among dental students and recruiting students from other dental schools may enhance the generalizability and representativeness of the sample. However, the findings of this study prove that although most respondents tend to have a good perception of SCD, formal education and continuous education through didactic learning and clinical training are still needed to prepare them and to increase their confidence and competence in providing care to special-needs patients. A dental school needs to participate in increasing SCD expertise through training and improving the SCD curriculum. Despite the study’s limitations, the results offer insights into areas that require further improvement, particularly in the provision of appropriate SCD education in Indonesia.

## 5. Conclusions

The present study indicates that these students tended to have good perceptions of SCD. Most clinical students are better able to work independently with special-needs patients following graduation and are more knowledgeable about SCD than preclinical students are. Most clinical students also need to refer special-needs patients to a specialist, and both preclinical and clinical students need and would consider postgraduate training in SCD to prepare for and to gain confidence and competence in providing care to special-care patients. This study suggests the need for a curriculum review, an evaluation of teaching materials and methods, clinical training for undergraduate and postgraduate students, and continuing education programs for SCD. This approach would result in a better understanding of SCD and enable students to respond appropriately to the growing number of special-needs patients.

## Figures and Tables

**Table 1:** Characteristics of the respondents, perception, and training programs related to SCD (N = 572).

Item	N (%)
Gender	
Male	84 (14.7)
Female	488 (85.3)
Study Program	
Preclinical	372 (65)
Clinical	200 (35)
Perception of Special-Needs Patients	
Have you treated any patients with special needs? (N = 200)	
No	120 (60)
Yes	80 (40)
What patient category have you treated? (N = 80)	
Elderly	62 (77.5)
Physically disabled	23 (28.8)
Intellectually disabled	20 (25)
Complex medical problems	16 (20)
Infectious disease	11 (13.8)
Psychological and behavioral disability	10 (12.5)
Blind	4 (5)
Deaf	3 (3.8)
I feel that I could work independently with patients with special needs following graduation.	
Strongly disagree	19 (3.3)
Disagree	142 (24.8)
Neutral	230 (40.2)
Agree	170 (29.7)
Strongly agree	11 (1.9)
Perception of Special-Care Dentistry	
Can you briefly define Special-Care Dentistry?	
No	164 (28.7)
Yes	408 (71.3)
Are you aware of Special-Care Dentistry?	
No	257 (44.9)
Yes	315 (55.1)
There is some controversy regarding the terminology for this new specialty, both nationally and internationally. Which title do you prefer?	
Special-Care Dentistry (SCD)	250 (43.7)
Special-Needs Dentistry (SND)	227 (39.7)
Unsure	95 (16.6)
How did you first hear about special-care dentistry?	
I have never heard of special-care dentistry	191 (33.4)
Through the internet	137 (24)
The academic staff informed me at my university/seminar	108 (18.9)
Through reading material (books/journals)	102 (17.8)
Through friends/relatives	32 (5.6)
Through the mass media (television/radio)	2 (0.3)
Training Program for Special-Care Dentistry	
Have you received clinical training for treating patients with special needs?	
No	537 (93.9)
Yes	35 (6.1)
The training that I received as a student is sufficient to prepare me for treating patients with special needs following graduation (N = 35)	
Strongly Disagree	0 (0)
Disagree	7 (20)
Neutral	9 (25.7)
Agree	15 (42.9)
Strongly Agree	4 (11.4)
Should your program have didactic teaching in Special-care Dentistry?	
No	25 (4.4)
Yes	547 (95.6)
In which years should your program consider didactic teaching in Special-Care Dentistry?	
First Year	128 (22.4)
Second Year	191 (33.4)
Third Year	340 (59.4)
Fourth Year	398 (69.6)
Fifth Year	352 (61.5)
Sixth Year	355 (62.1)
Should your program have clinical teaching in Special-Care Dentistry?	
No	35 (6.1)
Yes	537 (93.9)
In which years should clinical teaching in Special-Care Dentistry be conducted?	
First Year	117 (20.5)
Second Year	144 (25.2)
Third Year	226 (39.5)
Fourth Year	334 (58.4)
Fifth Year	413 (72.2)
Sixth Year	415 (72.6)
Do you think your program should have postgraduate training?	
No	59 (10.3)
Yes	513 (89.7)
Would you consider postgraduate training in Special-Care Dentistry if you had the opportunity?	
No	103 (18)
Yes	469 (82)

**Table 2:** Distribution of respondents’ perceptions of comfort in providing treatment to special-needs patients (N = 572).

Special-Needs Patients	Response N (%)					Mean ± SD
	Very Uncomfortable	Uncomfortable	Neutral	Comfortable	Very Comfortable	
Elderly	19 (3.3)	22 (3.8)	139 (24.3)	346 (60.5)	46 (8)	3.6 ± 0.8
Physical disability	18 (3.1)	23 (4)	173 (30.2)	303 (53)	55 (9.6)	3.6 ± 0.8
Intellectual disability	18 (3.1)	68 (11.9)	244 (42.7)	220 (38.5)	22 (3.8)	3.2 ± 0.8
Medically complex problem	21 (3.7)	66 (11.5)	208 (36.4)	255 (44.6)	22 (3.8)	3.3 ± 0.8
Infectious disease	38 (6.6)	159 (27.8)	251 (43.9)	116 (20.3)	8 (1.4)	2.8 ± 0.8
Behavioral or psychological problem	20 (3.5)	112 (19.6)	217 (37.9)	196 (34.3)	27 (4.7)	3.1 ± 0.1
Visual impairment	17 (3)	22 (3.8)	138 (24.1)	315 (55.1)	80 (14)	3.7 ± 0.8
Hearing impairment	21 (3.7)	54 (9.4)	186 (32.5)	257 (44.9)	54 (9.4)	3.4 ± 0.9

**Table 3:** Distribution of respondents’ perceptions of referring special-needs patients to specialists (N = 572).

Criteria Considered when Referring Special Need Patients to a Specialist	Response N (%)					Mean ± SD
	Never	Almost Never	Sometimes	Often	Always	
I would like a second opinion	10 (1.7)	22 (3.8)	270 (47.2)	201 (35.1)	69 (12.1)	3.5 ± 0.8
I am uncomfortable performing the necessary procedure	34 (3.8)	146 (15.9)	279 (48.8)	91 (25.5)	22 (5.9)	3.1 ± 0.8
I am unaware of how to proceed with treatment for a patient who is medically compromised	23 (4)	98 (17.1)	286 (50)	117 (20.5)	48 (8.4)	3.1 ± 0.9
The patient has an intellectual disability	36 (6.3)	116 (20.3)	273 (47.7)	112 (19.6)	35 (6.1)	2.9 ± 0.9
The patient has a physical disability	42 (7.3)	154 (26.9)	267 (46.7)	82 (14.3)	27 (4.7)	2.8 ± 0.9
The patient has psychological issues which preclude dental surgery	16 (2.8)	72 (12.6)	257 (44.9)	164 (28.7)	63 (11)	3.3 ± 0.9
The patient has behavioral issues which make treatment delivery difficult	19 (3.3)	65 (11.4)	247 (43.2)	183 (32)	58 (10.1)	3.3 ± 0.9
I have no disabled-friendly facilities	30 (5.2)	59 (10.3)	195 (34.1)	177 (30.9)	111 (19.4)	3.4 ± 1.0

**Table 4:** Respondents’ perceptions of special-care dentistry related to the study program (N = 572) and caring experience for special-needs patients (N = 200).

Items	Study Program			Clinical-Student Caring Experience for Patients with Special Needs		
	Preclinical, N (%) (N = 357)	Clinical, N (%) (N = 200)	*p* Value	No, N (%) (N = 120)	Yes, N (%) (N = 80)	*p* Value
Perception of special-needs patient						
Perception of being able to work independently with patients who have special needs following graduation (Mean ± SD) ^a^	3.0 ± 0.8	3.1 ± 0.8	<0.001 *	2.7 ± 0.8	2.9 ± 0.8	0.070
Perception of comfort toward providing treatment for special-needs patients (Mean ± SD) ^a^	3.3 ± 0.7	3.3 ± 0.5	0.313	3.3 ± 0.6	3.3 ± 0.5	0.845
Perception of referring special-needs patients to specialists (Mean ± SD) ^a^	3.2 ± 0.4	3.3 ± 0.5	0.042 *	3.2 ± 0.5	3.2 ± 0.5	0.301
Perception of special-care dentistry						
Assumption of knowledge about special-care dentistry ^b^			0.008 *			0.449
Yes	233 (62.6)	148 (74)		86 (71.7)	62 (77.5)	
No	139 (37.4)	52 (26)		34 (28.3)	18 (22.5)	
Can you briefly define special-care dentistry? ^b^			0.056			0.563
Yes	255 (68.5)	153 (76.5)		94 (78.3)	59 (73.8)	
No	117 (31.5)	47 (23.5)		26 (21.7)	21 (26.3)	
Are you aware of special-care dentistry? ^b^			0.811			0.839
Yes	203 (54.6)	112 (56)		66 (55)	46 (57.5)	
No	169 (45.4)	88 (44)		54 (45)	34 (42.5)	
Training program for special-care dentistry						
Have you received clinical training for treating patients with special needs? ^b^			0.233			0.558
Yes	19 (5.1)	16 (8)		8 (6.7)	8 (10)	
No	353 (94.9)	184 (92)		112 (93.3)	72 (90)	
Should your program have didactic teaching in special-care dentistry? ^b^			1.000			0.444
Yes	356 (95.7)	191 (95.5)		113 (94.2)	78 (97.5)	
No	16 (4.3)	9 (4.5)		7 (5.8)	2 (2.5)	
Should your program have clinical teaching in special-care dentistry? ^b^			0.054			0.880
Yes	355 (95.4)	182 (91)		110 (91.7)	72 (90)	
No	17 (4.6)	18 (9)		10 (8.3)	8 (10)	
Do you think your program should have postgraduate training? ^b^			<0.001 *			0.729
Yes	347 (93.3)	166 (83)		101 (84.2)	65 (81.3)	
No	25 (6.7)	34 (17)		19(15.8)	15 (18.8)	
Would you consider postgraduate training in special-care dentistry if you had the opportunity? ^b^			0.004 *			0.298
Yes	318 (85.5)	151 (75.5)		87 (72.5)	64 (80)	
No	54 (14.5)	49 (24.5)		33(27.5)	16 (20)	

^a^ Mann–Whitney test; ^b^ Chi-square test; * Significance, *p* value < 0.05.

## Data Availability

The dataset used in the current study is available upon request from the corresponding author.
